# Supplementary material for: Tryptophan metabolite atlas uncovers organ, age, and sex-specific variations
Source: bioRxiv. 2024 Dec 23:2024.12.23.630041. Preprint. [Version 1] doi: 10.1101/2024.12.23.630041 (PMC11703250; doi:10.1101/2024.12.23.630041)
Supplement: 1 [file NIHPP2024.12.23.630041V1-supplement-1.pdf]

bioRxiv preprint doi: https://doi.org/10.1101/2024.12.23.630041; this version posted December 23, 2024. The copyright holder for this preprint (which was not certified by peer review) is the author/funder, who has granted bioRxiv a license to display the preprint in perpetuity. It is made available under aCC-BY 4.0 International license.

SUPPLEMENTAL FIGURE LEGENDS

Figure S1: Control curves and metabolite abundance in mice under varying diets analyzed by LC-MS/MS.

(A-D) Control curves per metabolite groups ran in the different columns by LC-MS/MS.

(E-H) Metabolite abundance (ng/mL) in mice under varying diets, measured through LC-MS/MS.

Figure S2: q-Values from sex comparison differences across age groups (3, 53, and 74 weeks) in various organs and tissues

(A-C) Tables displaying significant and near-significant q-values from sex comparison differences, determined by multiple t-tests, across age groups: 3 weeks (A), 53 weeks (B), and 74 weeks (C), for various organs and tissues.

Figure S3: q-Values from sex comparison differences across age groups (3, 53, and 74 weeks) in various brain regions.

(A-C) Tables displaying significant and near-significant q-values from sex comparison differences, determined by multiple t-tests, across age groups: 3 weeks (A), 53 weeks (B), and 74 weeks (C), for various brain regions.

Figure S4: Trp metabolites in different diets.

(A-E) Measurement of the concentrations of various Trp metabolites using LC-MS/MS across three distinct diets: Chow (standard diet for mice), AA (controlled amino acid-sufficient diet), and TF (tryptophan-deficient amino acid diet).

bioRxiv preprint doi: https://doi.org/10.1101/2024.12.23.630041; this version posted December 23, 2024. The copyright holder for this preprint (which was not certified by peer review) is the author/funder, who has granted bioRxiv a license to display the preprint in perpetuity. It is made available under aCC-BY 4.0 International license.

Table S1. Trp metabolite content in Chow, defined amino acid diet (AA) compared to Trp-free diet (TF) measured by LC-MS/MS

**Metabolites**	**Diets**		
	**Chow** (ng/g)	**AA** (ng/g)	**TF** (ng/g)
Trp	190800.5	1474940.5	393.3
NFK	195.3	552.8	11.0
Kyn	2871.0	5260.8	66.0
KA	308.0	3627.3	8.3
AA	132.0	19.3	2.8
NFAA	0	0	0
XA	147.4	188.9	191.1
3HAA	0	0	0
CA	3137.8	649.0	30.3
I3P	141531.5	3250.5	2475.0
ILA	858.0	324.5	33.0
I3A	1248.5	522.5	11.0
5HTP	90.8	214.5	2.8
Serotonin	2035.0	1534.5	409.8
5HIAA	6272.8	211.8	115.5
Melatonin	11.0	0	0
Tryptamine	3778.5	1193.5	88.0

bioRxiv preprint doi: https://doi.org/10.1101/2024.12.23.630041; this version posted December 23, 2024. The copyright holder for this preprint (which was not certified by peer review) is the author/funder, who has granted bioRxiv a license to display the preprint in perpetuity. It is made available under aCC-BY 4.0 International license.

A

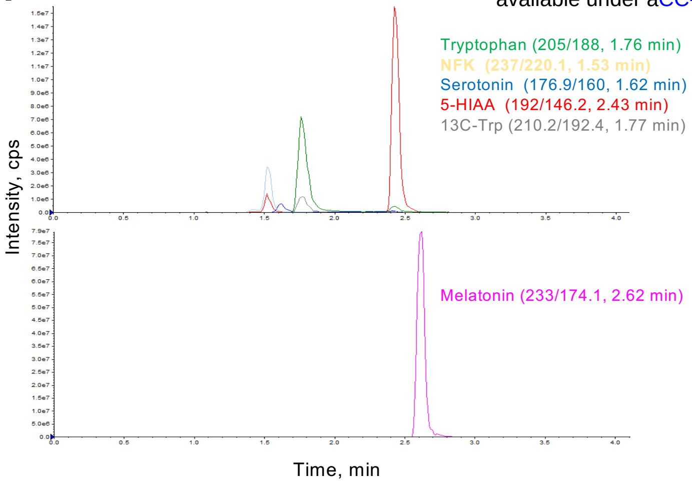

Tryptophan, NFK (N-formyl kynurenine), Serotonin, 5-HIAA (5-hydroxyindoleacetic acid), 13C-Tryptophan, and Melatonin, 1000 ng/mL each, run together in a standard curve.

B

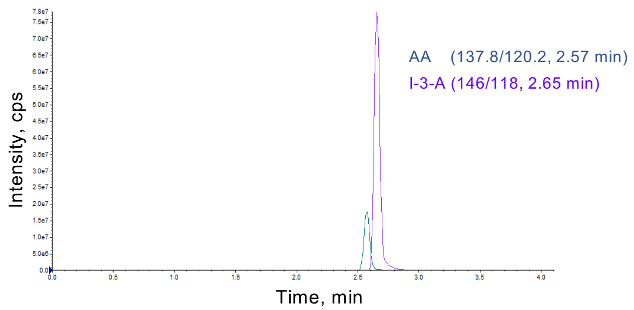

AA (Anthranilic acid) and I-3-C (Indole-3-carboxyaldehyde), 100 ng/mL each, run together in a standard curve

C

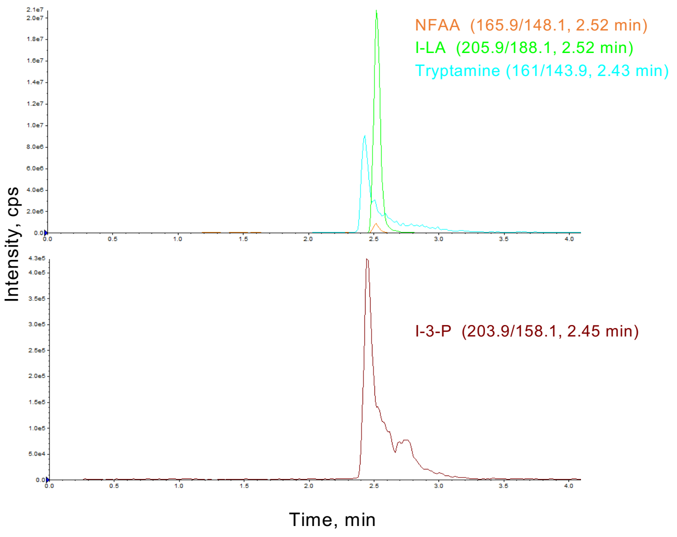

NFAA (N-formylanthranilic acid), I-LA (Indole-3-lactic acid), and Tryptamine, 100 ng/mL each; I-3-P (Indole-3-pyruvic acid), 1000 ng/mL, run together in a standard curve

D

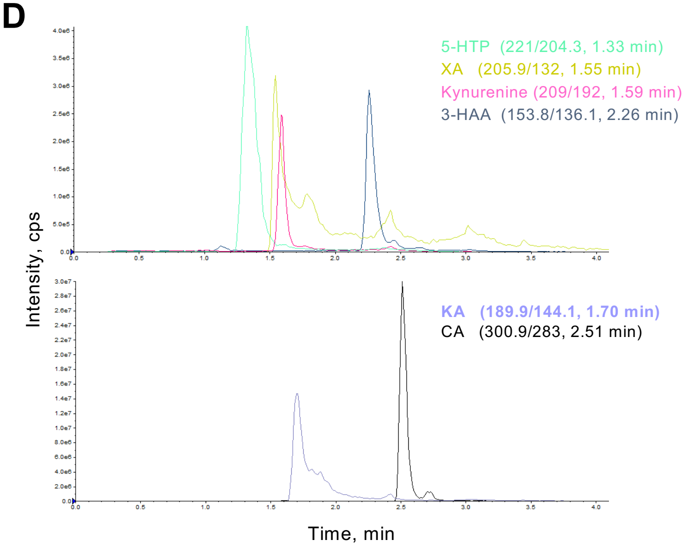

Kynurenine, 3-HAA (3-hydroxyanthranilic acid), XA (Xanthurenic acid), and 5-HTP (5-hydroxytryptophan), 5000ng/mL each; KA (Kynurenic acid) and CA (Cinnabarinic acid), 1000 ng/mL each, run together in a standard curve

Figure S1

bioRxiv preprint doi: https://doi.org/10.1101/2024.12.23.630041; this version posted December 23, 2024. The copyright holder for this preprint (which was not certified by peer review) is the author/funder, who has granted bioRxiv a license to display the preprint in perpetuity. It is made available under aCC-BY 4.0 International license.

A

Significant Sex Differences Across Tissues (3 weeks)

3 w FvM	Tryptophan	Kynurenine	NFK	5HIAA	I3P	CA	KA	XA	I3A	Serotonin	AA	ILA	Tryptamine	5-HTP
Colon														
Heart														
Lung														
Spleen		0.07782												
Kidney								0.03719						
Liver													0.05959	
Gonads	0.02269	0.00657			0.00032									
BAT														
Muscle														
Thymus														
ingWAT	0.0009								0.0711					
Serum														

B

Significant Sex Differences Across Tissues (53 weeks)

53 w FvM	Tryptophan	Kynurenine	NFK	5HIAA	I3P	CA	KA	XA	I3A	Serotonin	AA	ILA	Tryptamine	5-HTP
Colon	0.04296													
Heart														
Lung												0.10707		
Spleen														
Kidney						0.1221		0.02115						
Liver					0.00073							0.1387		
Gonads	0.02269				0.02607							0.02915		
BAT														
Muscle		0.07534												
Thymus														
ingWAT	0.0007													
Serum	0.06377	0.03655		0.00262	0.06556				0.01293			0.00365		

C

Significant Sex Differences Across Tissues (74 weeks)

74 w FvM	Tryptophan	Kynurenine	NFK	5HIAA	I3P	CA	KA	XA	I3A	Serotonin	AA	ILA	Tryptamine	5-HTP
Colon														
Heart						0.01012								
Lung			0.09648					0.05807						
Spleen														
Kidney			0.02272						0.10343					
Liver		0.00025			0.00151	0.09099			0.00128				0.00246	
Gonads					0.00312				0.11882					
BAT														
Muscle						0.12176								
Thymus														
ingWAT														
Serum	0.06377								0.01293			0.01581		0.069<br>21

Legend:

- Not significant
- Significantly Higher in females
- Significantly Higher in males

Figure S2

bioRxiv preprint doi: https://doi.org/10.1101/2024.12.23.630041; this version posted December 23, 2024. The copyright holder for this preprint (which was not certified by peer review) is the author/funder, who has granted bioRxiv a license to display the preprint in perpetuity. It is made available under aCC-BY 4.0 International license.

A

Significant Sex Differences Across Tissues (3 weeks)

**Brainstem**	0.045544							0.03623	
**Cerebellum**						0.03354		0.01642	
**Diencephalon**									
**Cortex**									
**3 w FvM**	Tryptophan	Kynurenine	NFK	5HIAA	I3P	CA	KA	XA	I3CA

B

Significant Sex Differences Across Tissues (53 weeks)

**Brainstem**						0.05605		0.01229	
**Cerebellum**									
**Diencephalon**									
**Cortex**				0.14986					
**53 w FvM**	Tryptophan	Kynurenine	NFK	5HIAA	I3P	CA	KA	XA	I3CA

C

Significant Sex Differences Across Tissues (74 weeks)

**Brainstem**	0.033888				0.03914			0.0534	
**Cerebellum**								0.01642	
**Diencephalon**								0.02032	
**Cortex**									
**74 w FvM**	Tryptophan	Kynurenine	NFK	5HIAA	I3P	CA	KA	XA	I3CA

Legend:

Not significant

Significantly Higher in females

Significantly Higher in males

Figure S3

bioRxiv preprint doi: https://doi.org/10.1101/2024.12.23.630041; this version posted December 23, 2024. The copyright holder for this preprint (which was not certified by peer review) is the author/funder, who has granted bioRxiv a license to display the preprint in perpetuity. It is made available under aCC-BY 4.0 International license.

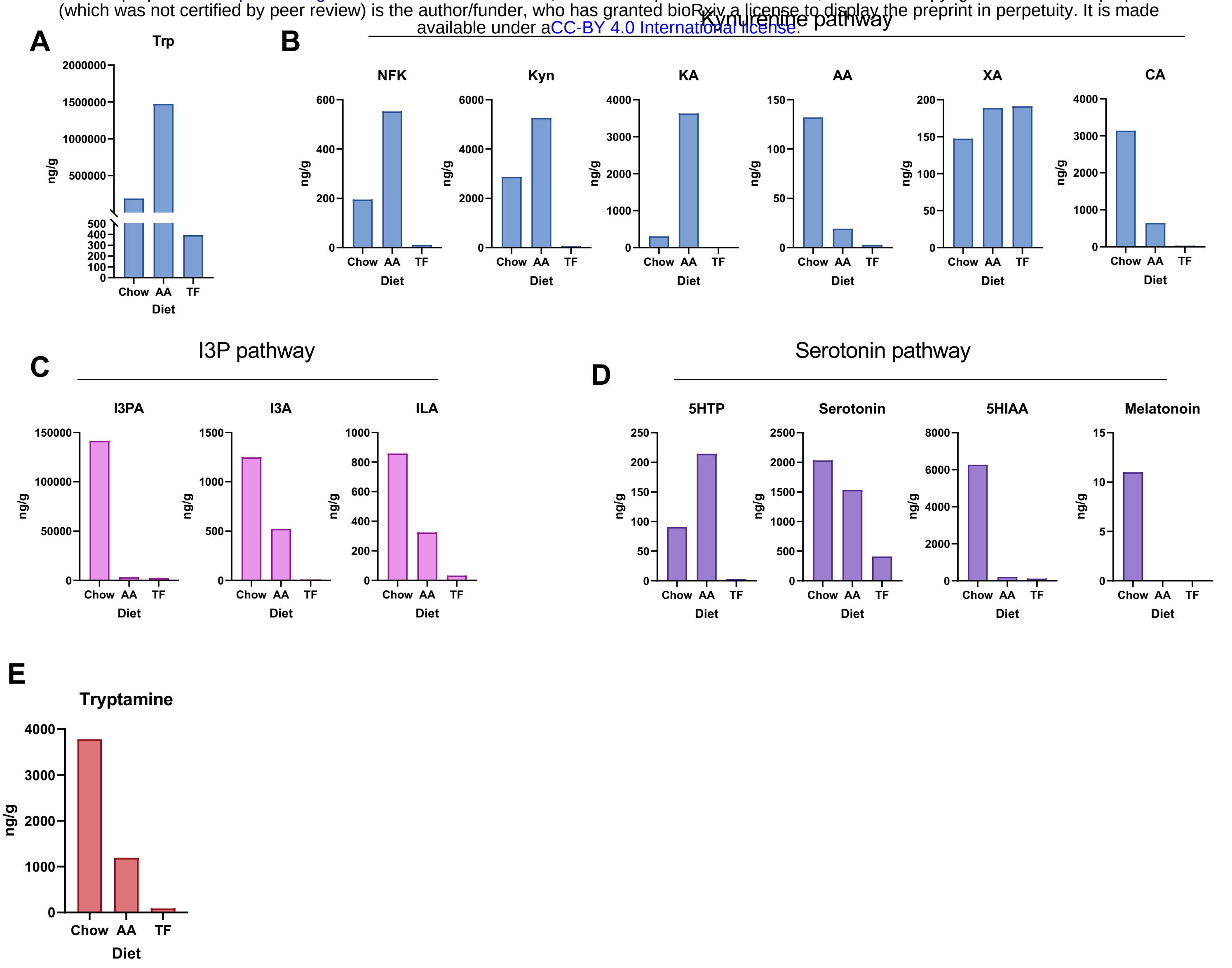

Figure S4
